# Improved Particle Filter Algorithm for Multi-Target Detection and Tracking

**DOI:** 10.3390/s24144708

**Published:** 2024-07-20

**Authors:** Yi Cheng, Wenbo Ren, Chunbo Xiu, Yiyang Li

**Affiliations:** School of Control Science and Engineering, Tiangong University, Tianjin 300387, China; chengyi@tiangong.edu.cn (Y.C.); xiuchunbo@tiangong.edu.cn (C.X.); yang1599999@gmail.com (Y.L.)

**Keywords:** particle filter, multi-target, density-based clustering, detection and tracking

## Abstract

In modern radar detection systems, the particle filter technique has become one of the core algorithms for real-time target detection and tracking due to its good nonlinear and non-Gaussian system state estimation capability. However, when dealing with complex dynamic scenes, the traditional particle filter algorithm exposes obvious deficiencies. The main expression is that the sample degradation is serious, which leads to a decrease in estimation accuracy. In multi-target states, the algorithm is difficult to effectively distinguish and stably track each target, which increases the difficulty of state estimation. These problems limit the application potential of particle filter technology in multi-target complex environments, and there is an urgent need to develop a more advanced algorithmic framework to enhance its robustness and accuracy in complex scenes. Therefore, this paper proposes an improved particle filter algorithm for multi-target detection and tracking. Firstly, the particles are divided into tracking particles and searching particles. The tracking particles are used to maintain and update the trajectory information of the target, and the searching particles are used to identify and screen out multiple potential targets in the environment, to sufficiently improve the diversity of the particles. Secondly, the density-based spatial clustering of applications with noise is integrated into the resampling phase to improve the efficiency and accuracy of particle replication, so that the algorithm can effectively track multiple targets. Experimental result shows that the proposed algorithm can effectively improve the detection probability, and it has a lower root mean square error (RMSE) and a stronger adaptability to multi-target situation.

## 1. Introduction

In recent years, particle filter algorithms have become a popular research direction in radar target detection and tracking. As a nonparametric filter algorithm based on random sampling, the particle filter algorithm has good adaptability and robustness. It is able to utilize radar measurements to estimate and predict the state of the target and achieve accurate target tracking [1]. The main steps of the particle filter algorithm are initialization, state updating, resampling, and state output. During the resampling process, replicating high-weight particles and eliminating low-weight particles, complex nonlinear dynamic properties or nonlinear observations make it difficult for particles to effectively cover the entire state space, which greatly reduces particle diversity. The resampling phase of the traditional particle filter algorithm leads to the concentration of the particle population in the region where the observed target signal is strongest. Therefore, enhancing the diversity of the particle population is important to improve the algorithm’s performance.

In order to overcome the problem of diversity degradation, researchers have proposed various improvements. Zhou et al. [2] optimized the particle filter algorithm by introducing an improved firefly algorithm, and the estimation accuracy was significantly improved, but the setting of multiple fixed parameters of the introduced firefly algorithm may increase the sensitivity of the algorithm to environmental changes, which reduces its applicability and generalization ability in diverse environments. Okuma et al. [3] proposed a multi-target tracking system combining a mixture of particle filters and the Adaboost algorithm. The system uses an HSV color histogram as an observation model to achieve stable tracking of athletes in the video. However, the performance of Adaboost is highly dependent on the quality and quantity of training data, and limited training data may lead to a decrease in detection accuracy. Kreucher et al. [4] proposed a particle filter approach to recursively estimating the joint multi-target probability density for simultaneous multi-target detection and tracking. However, the proposed method does not reflect the timely detection and tracking of midway nascent targets. Wang et al. [5] introduced the concept of successive-target-cancellation to simplify the joint search process of multi-target states into multiple independent single-target detection processes, which effectively improves the detection probability of multi-targets. Úbeda-Medina et al. [6] proposed an adaptive filter that combines two types of particle filters: the target resampling auxiliary particle filter and the auxiliary particle filter. This allows the algorithm to track an arbitrary number of targets and still have a high-reliability performance, but it is only suitable for situations where the number of targets is fixed and known. Chen et al. [7] proposed an improved multi-motorized target tracking algorithm based on a novel intelligent particle filter algorithm. This algorithm combines the bat algorithm and particle filter algorithm, using the latest observed value to intelligently move particles to a more optimal position in the global region, thereby improving the accuracy of target tracking. Tian et al. [8] improved the estimation accuracy of the target state by treating the particles as fireflies and using a spring model to guide other particles toward the optimal particle. Jiang et al. [9] introduced a genetic algorithm to simulate the selection, crossover, and mutation of genes in nature by using the selection, crossover, and mutation of particles, which effectively solved the phenomenon of particle degradation. Liu et al. [10] integrated the measurement noise into a Gaussian-like probability distribution to obtain an accurate measurement likelihood, increasing the diversity of particles and using particle swarm optimization techniques to reduce computational complexity. Hu et al. [11] introduced an extended Kalman filter and genetic algorithm into the particle filter to improve the sampling accuracy and effectiveness of the particle probability density function, which effectively ensures the diversity of particle states in the particle swarm. However, these algorithms have limited their adaptability in coping with multi-target situations.

For multi-target detection and tracking methods based on other algorithms, Shamsfakhr et al. [12] proposed a multi-target detection and position tracking algorithm based on mmWave-FMCW radar data, using an effective clustering approach and by a final Structured Branching Multiple Hypothesis Testing algorithms. The algorithm realizes the accurate detection and tracking of multi-target in indoor environments. However, the experiments of the proposed algorithm were conducted in a specific indoor environment and the results may not apply to other different environments. Zhu et al. [13] proposed a candidate-plots-based DP-TBD (CP-DP-TBD) method, which provides candidate graphs with target loss detection information through an improved merit function transfer procedure. However, when the state space and decision space become exceedingly large, the computational cost escalates dramatically, leading to the notorious issue known as the “curse of dimensionality”. Üney et al. [14] proposed a Bayesian detection method based on the Bernoulli state-space model by integrating the fuzzy function of the radar data cube over a long time and realizing the effective detection of targets with low signal-to-noise ratio. Liang et al. [15] proposed a BP method for track-before-detect. The method introduces a new statistical model, develops the corresponding factor graph, and performs inference by applying BP on that graph.

The existing particle filter-based detection and tracking algorithms still suffer from diversity degradation, which diminishes algorithms’ ability to distinguish multiple targets. In addition, when dealing with multi-target cases where the number of targets is unknown and the number of targets varies randomly, the detection ability of the existing algorithms is greatly reduced compared to a single-target case. To address the above situation, this paper proposes a multi-target detection and tracking method based on an optimized particle filter algorithm. Firstly, the particles are divided into tracking particles and searching particles to maximize the diversity of particles. Secondly, the density-based spatial clustering of applications with noise (DBSCAN) [16] algorithm is used to optimize the resampling step. This optimization method changes the traditional particle replication method to a replication method that can take into account the effective particles of multi-target at the same time. This method also changes the original scheme of measuring the magnitude of particle weights to measuring whether the target particles exist, enhancing the detection and tracking ability of multi-target, so that the algorithm is more adaptable to multi-target detection and tracking.

The organizational structure of the remaining sections of the paper is delineated as follows. In Section 2, the flow of the traditional particle filter algorithm and its problems are presented and are introduced. Section 3 introduces the basic principles and algorithmic flow of the DBSCAN algorithm. Section 4 describes the improved multi-target particle filter detection and tracking algorithm and the overall flow of the algorithm. Section 5 verifies the effectiveness of the improved algorithm through simulation and comparison experiments. Section 6 summarizes the improvement ideas of the algorithm in this paper and points out the significance of the improved algorithm.

## 2. Particle Filter Algorithm

The particle filter algorithm combines sequential importance sampling (SIS) and resampling, using the importance sampling density function $q(x_{k}|z_{1:k})$ to extract samples, where $x_{k}$ and $z_{k}$ denote the state vector and the observation vector at the moment k. The particle filter algorithm selects the prior probability $p(x_{k}|x_{k-1}^{i})$ as the importance density function to update the weights [17,18], using weighted particles to represent the conditional posterior probability density $p(x_{k}|z_{1:k})$ of the system state. The specific steps of the particle filter algorithm can be summarized as follows:

1. Particle Initialization;

Generating N particles within the observation region is based on the initial probability density $p(x_{0})$, where $p(x_{0})$ is uniformly distributed in the observation region, resulting in an initial particle swarm ${\left\{x_{0}^{i},w_{0}^{i}\right\}}_{i=1}^{N}$, where $x_{0}^{i}$ denotes the state of the ith particle and $w_{0}^{i}$ denotes the weight corresponding to the state of the *i*th particle.

2. State Updating;

Particles from the previous frame are introduced for state transition, followed by importance weight calculation and normalization processing. The particle importance weights are calculated in the following equation:

(1) $${\tilde{w}}_{k}^{i}=w_{k-1}^{i}l(z_{k}|x_{k}^{i},E_{k}=e_{k}),i=1,2,\cdots N$$

where l denotes the likelihood ratio of the particle.

Normalizing the obtained particle importance weights and calculating are as follows:

(2) $$w_{k}^{i}=\frac{{\tilde{w}}_{k}^{i}}{\sum_{j=1}^{N}{\tilde{w}}_{k}^{j}},i=1,2,\cdots N$$

3. Resampling;

Comparing the threshold value *N*th with the effective number of particles *N_eff_*. if *N*_th_ < *N_eff_*, performing a resampling operation on the normalized particles by discarding the small-weighted particles, copying the large-weighted particles, and setting the weights of all the particles to $w_{k}^{i}=1/N$ to obtain a new set of particles ${\left\{x_{k}^{i},w_{k}^{i}\right\}}_{i=1}^{N}$. where:

(3) $$N_{eff}=\frac{1}{\sum_{i=1}^{N}{\left(w_{k}^{i}\right)}^{2}}$$

4. State Output;

Approximating the current state value by weighting the sampled particles:

(4) $${\hat{x}}_{k}=\sum_{i=1}^{N}w_{k}^{i}x_{k}^{i}$$

5. Let k=k+1 and return to step 2 for the next moment of state estimation until the end of filtering.

**Figure 1:**
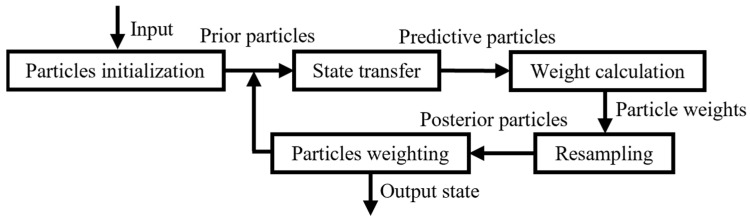
Particle filter algorithm flowchart.

Figure 1 shows the flowchart of the particle filter algorithm. The resampling mechanism of the particle filter algorithm will inevitably appear particle degradation phenomenon. After multiple iterations, the resampling stage will concentrate the particles in the vicinity of the target with the highest signal-to-noise ratio, resulting in serious missed detections when dealing with the problem of multi-target detection and tracking [19,20,21]. Therefore, optimizing the resampling process is necessary to improve the particle diversity.

## 3. Density-Based Spatial Clustering of Applications with Noise

DBSCAN is a data mining algorithm for cluster analysis, which can discover clusters of arbitrary shapes and identify noisy data points [22,23,24,25,26]. Unlike clustering algorithms such as K-Means and BIRCH, which are generally applicable only to convex sample sets, DBSCAN can be applied to both convex and non-convex sample sets. The DBSCAN algorithm performs clustering based on the density of the sample points without the need to pre-set the number of clusters. Therefore, it has high flexibility in practical applications.

The main idea of the DBSCAN algorithm is to divide regions with sufficient density into clusters and to discover arbitrarily shaped clusters in a spatial database with noise. The parameters and related concepts involved in the algorithm are elaborated below:

**Definition 1 (**Eps **Neighborhood).**  *The set of all points in the region centered at any point* e*, with Eps as the radius, i.e.:*

(5) $$N_{Eps}(e)=\{g\in D||dist(e,g)\leq Eps\}$$

*where D is the sample data set and* dist(e,g) *is the distance between point* e *and point* g.

**Definition 2 (Minimum number** MinPts**).**  *The key parameter for determining whether any point* e *can be defined as a core point. A point* e *is considered to be a core point if it contains at least* MinPts *samples in its Eps neighborhood.*

**Figure 2:**
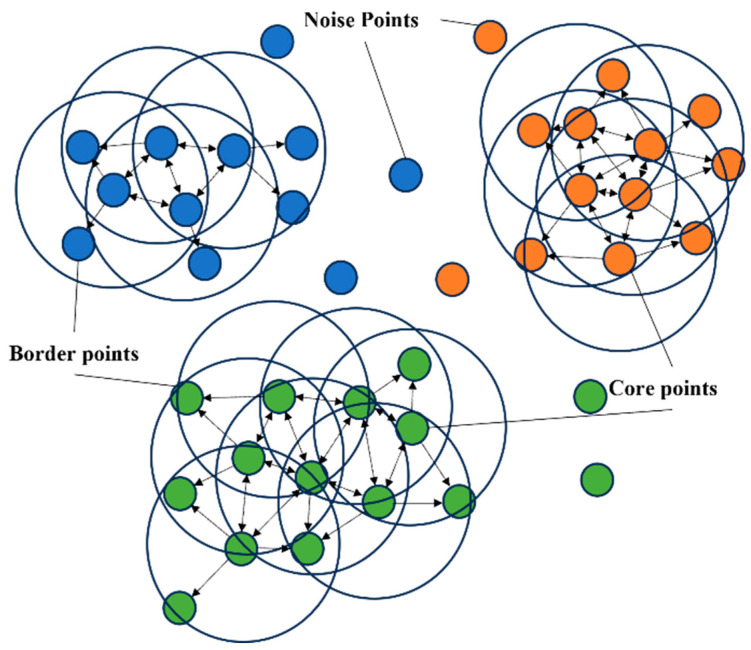
Searching for core points and forming temporary clusters.

**Definition 3 (Core, Border, and Noise Points).**  *As shown in* Figure 2*, for any point* e*, if* $\left|N_{Eps}(e)\right|>MinPts$*, then* e *is defined as a core point; if the point* e *lies in the* Eps *neighborhood of some core point but does not satisfy the condition of being a core point itself, then* e *is defined as a border point; if the point* e *does not lie in the* Eps *neighborhood of any core point, then* e *is defined as a noise point.*

**Definition 4 (Directly density-reachable).**  *For any core point* e*, if any point* g *is located in the* Eps *neighborhood of point* e*, then point* e *is defined as being directly density reachable from point* g.

**Definition 5 (Density-reachable).**  *If there exist points* $e_{1},\;e_{2},\;\cdots e_{n}\in D$ *where point* $e_{i+1}$ *is directly density reachable from point* $e_{i}$*, then point* $e_{n}$ *is defined to be directly reachable from point* $e_{1}$.

**Definition 6 (Density-connected).**  *If there is a point* o∈D *where point* e *and point* g *are density reachable from point* o*, then point* e *and point* g *are defined as density connected.*

**Definition 7 (Cluster).**  *A cluster is a set of all density-connected points. A set* C *is defined to be a cluster if there exists* $g_{1},\;g_{2},\;\cdots g_{n}\in D$ *and any two of the points* $g_{i}$ *and* $g_{j}$ *are density connected.*

Algorithm process:

1. Take the unprocessed points from the database and calculate the neighborhood of any point e based on $dist\left(e,g\right)=\sqrt{{\left(x_{e}-x_{g}\right)}^{2}+{\left(y_{e}-y_{g}\right)}^{2}}$, denoted as $N_{Eps}(e)$.
2. Determine the type of the point, if the point is a core point, using the given domain as the density reachable distance to find the density reachable object of the core point, and then forming a temporary cluster; if the point is a border point, it is categorized as a temporary clustering cluster with its nearest core point belonging to the cluster; if the point is a noise point, then outputting the point separately as an outlier.
3. Merge temporary clusters to obtain clusters.
4. Repeat operations 1, 2, and 3 until all generated clusters meet the density requirement.

Figure 2 shows the core, border, and noise points, as well as the correlation between the points and the process of forming temporary clustering clusters based on the correlation between the points.

## 4. Improved Multi-Target Particle Filter Detection and Tracking Algorithm

**Figure 3:**
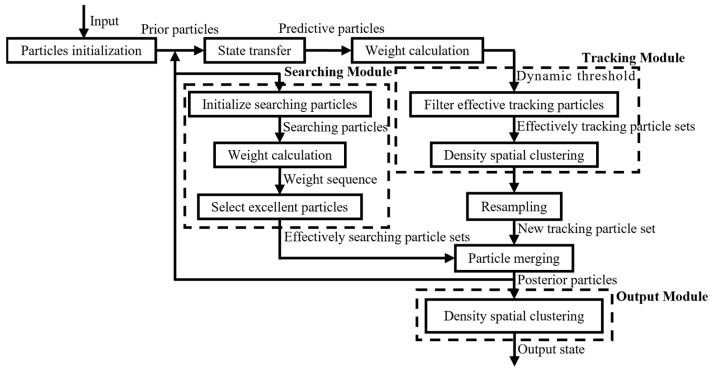
Improved PF algorithm flowchart.

Aiming at the defect of particle diversity degradation in particle filter algorithm for detecting and tracking multi-target, to improve the particle diversity, this paper divides the particles into tracking particles and searching particles, using the DBSCAN algorithm to optimize the particle filter process. The improved algorithm schematic block flowchart is shown in Figure 3.

The algorithmic improvements and the overall process are described as follows:

1. Particle Initialization

Generate $N_{t}$ particles in the observation area according to the initial probability density $p(x_{0})$, where $p(x_{0})$ is uniformly distributed within the observation area, thus obtaining the initial particle swarm ${\left\{x_{0}^{i},\;w_{0}^{i}\right\}}_{i=1}^{N_{t}}$.

2. State Transfer and Particle Testing

Introduce the particle from the previous frame, perform state transition, and calculate the importance weight. The particle importance weight is calculated as follows:

(6) $${\tilde{w}}_{k}^{i}=\frac{1}{N}l(z_{k}|x_{k}^{i},E_{k}=e_{k}),i=1,2,\cdots N_{t}$$

Normalize the obtained particle importance weights and calculate as follows:

(7) $$w_{k}^{i}=\frac{{\tilde{w}}_{k}^{i}}{\sum_{j=1}^{N}{\tilde{w}}_{k}^{j}},i=1,2,\cdots N_{t}$$

3. Filter effective tracking particles

During resampling, the particles at this time will be selected and copied. However, for new targets in the middle and targets that cannot be accurately tracked at this time, particle degradation inevitably occurs, and the tracking effect is poor. Here, particles are divided into tracking particles and searching particles, with tracking particles as the main and searching particles as the auxiliary, to maintain the diversity of particles.

The dynamic threshold T is first generated from the predicted particle weights at time k. The dynamic threshold is then used to filter the set of effective tracking particles ${\left\{{\hat{x}}_{k}^{i}\right\}}_{i=1}^{N_{et}}$ from the set of predicted particles ${\left\{x_{k}^{i},\;w_{k}^{i}\right\}}_{i=1}^{N_{t}}$. The dynamic threshold and effective tracking particle set are calculated as follows:

(8) $$T=10^{\left(\mathrm{lg}w_{k,\max}+\mathrm{lg}w_{k,\min}\right)}$$

(9) $${\left\{{\hat{x}}_{k}^{i}\right\}}_{i=1}^{N_{et}}=\{x_{k}^{i}\in {\left\{x_{k}^{i},w_{k}^{i}\right\}}_{i=1}^{N_{et}}:w_{k}^{i}>T\}$$

4. Density Spatial Clustering

Since the weights of particles conforming to the same target state are similar, and the weights of invalid particles (the distribution law is consistent with the noise distribution law) are widely dispersed, clustering can be used to screen out the effective particles that have successfully tracked the target. If there is an outlier in the effective particle set, it is most likely that the outlier is a target not represented in the original particle set. The outlier comes from the search particle or the state transition of the old particle swarm, so it is necessary to increase the representation proportion of the target in the particle set. The DBSCAN clustering algorithm can achieve the above operations. The DBSCAN algorithm cannot only distinguish each particle population and get the pseudo-target number but also separate each outlier. Compared with other clustering algorithms, the DBSCAN algorithm accurately categorizes all valid particles and is relatively easy to implement, which makes it extremely effective in coping with the problem of detecting and tracking multi-target.

The set of effectively tracked particles will be clustered using the DBSCAN algorithm. At this time, the clustering result set of all tracked targets will be obtained. If there are outliers in the clustering result, each outlier is assigned a weight value of $1/n_{p}$, where $n_{p}$ is the number of outliers, the remaining particles are given the same weight $1/N_{t}$; if there are no outliers, all particles are given the same weight $1/N_{t}$.

5. Resampling

Mapping in proportion to the particle weights and reserving $N_{es}$ seats for holding effectively searching particles to get a new set of tracking particles ${\left\{x_{k+1}^{i},\;w_{k+1}^{i}\right\}}_{i=1}^{N_{t}-N_{es}}$.

6. Searching with particles

A new batch of particles satisfying the initial probability density distribution is generated in each frame as search particles. To improve the diversity of particles and reduce the amount of calculation, the number of searching particles is set significantly lower than the number of tracking particles. Then the first $N_{es}$ particles with larger weights are selected according to the weights.

7. Particle merging

Merging two kinds of particles yields the set of tracking particles ${\left\{x_{k+1}^{i},\;w_{k+1}^{i}\right\}}_{i=1}^{N_{t}}$ at time k.

8. State output

In the state output phase, when the system is dealing with a multi-target situation, the output particle set contains multiple target information, so it is not applicable to approximate the current state value by particle weighting. The DBSCAN algorithm is used to process the output particle set, which can clearly and accurately obtain the target state corresponding to each particle group, and the clustering result is not affected by the number of targets. The merged posterior particles representing the target state at this time are clustered again by the DBSCAN algorithm, and the clustering result is all the target state values at the current time.

## 5. Experimentation and Analysis

In order to verify the algorithm’s effectiveness, simulation experiments were conducted to compare it with the traditional particle filter algorithm, the particle filter algorithm optimized based on the firefly algorithm (FA-PF), and the particle filter algorithm based on successive target cancellation (STC-PF).

### 5.1. Experimental Parameters and Preprocessing

Radar echo data of 6400 pulses are generated from the radar parameters simulation shown in Table 1, where each pulse data contains 1050 range cells. Each 64 pulses are treated separately as a CPI, and the number of pulses in a single CPI is set to 64, totaling 100 CPIs. To intuitively display the moving target in the range dimension, MTD processing is carried out after the pulse compression processing of the echo data in units of each CPI. Finally, the maximum pulse repetition frequency is extracted to obtain 100 frames of echo data, which is equivalent to extracting the effective information of every 64 pulses of echo data into a single frame. The target parameters in the echoes are shown in Table 2, where the target moves with uniform or uniformly variable speed and appears at different moments. The RMSE and the probability of target presence are used as performance evaluation indicators, and the RMSE distance parameter is set to truncate the distance to 50m.

### 5.2. Comparative Analysis of Target Presence Probability

**Figure 4:**
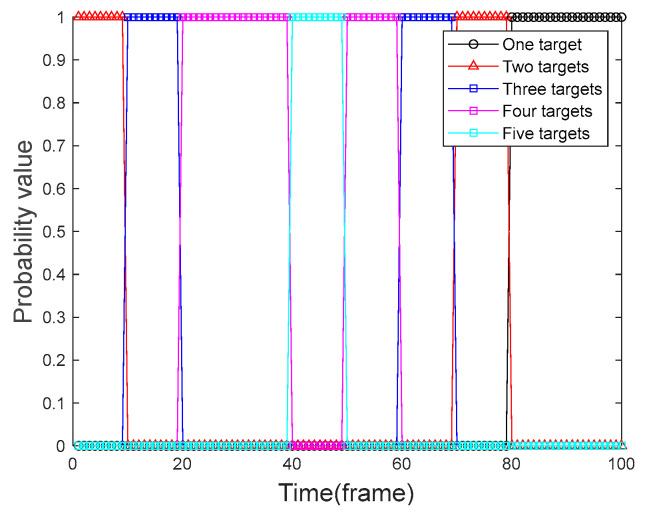
Probability graph of the true presence of the target.

**Figure 5:**
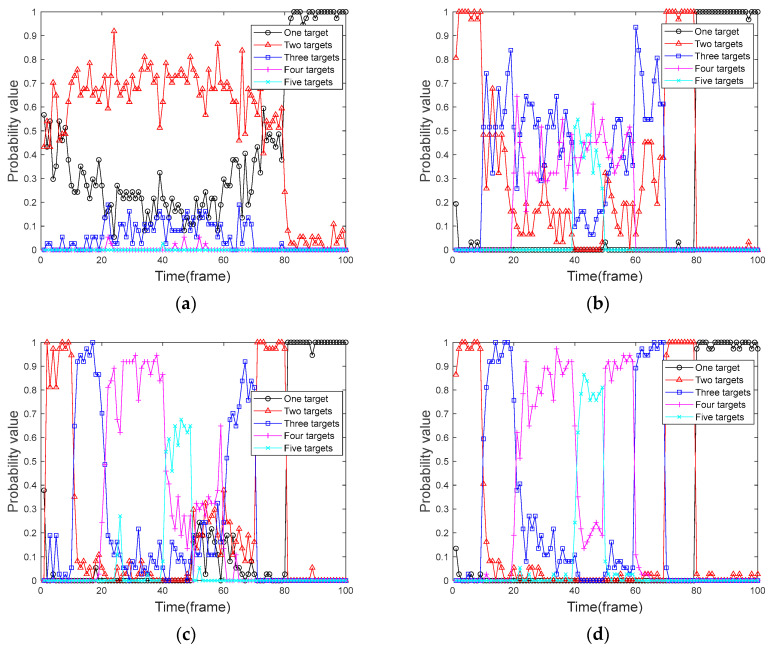
Probability of uniformly moving target presence. (**a**) Traditional PF algorithm; (**b**) FA-PF algorithm; (**c**) STC-PF algorithm; (**d**) Improved the PF algorithm.

**Figure 6:**
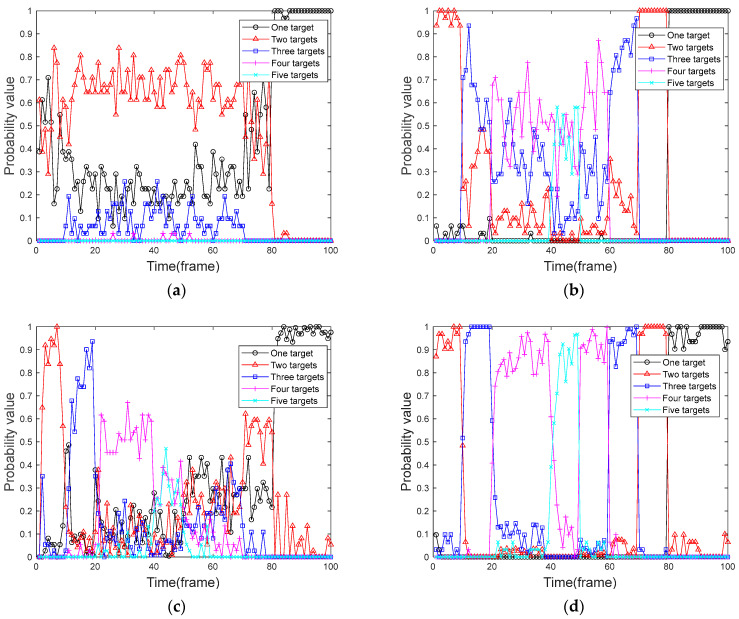
Probability of uniformly accelerated target presence. (**a**) Traditional PF algorithm; (**b**) FA-PF algorithm; (**c**) STC-PF algorithm; (**d**) Improved the PF algorithm.

We used four algorithms to perform frame-by-frame detection on the 100 frames of data obtained in Section 5.1. We designed and executed 100 Monte Carlo simulation experiments to calculate the probability of the target presence graph of the corresponding algorithm by the number of targets obtained in each frame. The target presence probabilities of the four algorithms are compared and analyzed. Figure 4 shows the graph of the real presence probability of the target. Figure 5 shows the probability of target presence for each algorithm in the context of a uniformly moving target, and Figure 6 shows the probability of target presence for each algorithm in the context of a uniformly accelerated target. Among them, Figure 5a and Figure 6a show the target presence probability graph of the traditional PF algorithm; Figure 5b and Figure 6b show the target presence probability graph of the FA-PF algorithm; Figure 5c and Figure 6c show the target presence probability graph of the STC-PF algorithm; and Figure 5d and Figure 6d show the target presence probability graph of the improved PF algorithm in this paper.

By comparing the target presence probability in the context of uniformly moving targets in Figure 5, it can be seen that in the single-target phase, i.e., frame 80 to frame 100, the missed detection probability of the four algorithms is extremely low; the target presence probability can reach more than 90%. In the multi-target stage, the traditional PF algorithm can almost only provide the state information of up to two targets simultaneously. The detection probability of the FA-PF algorithm is better than that of the traditional PF algorithm, and it has a certain ability to cope with the multi-target situation where the probability of the three targets existing at the same time can be more than 50%; however, it is unable to cope with the situation that the number of targets is more. The STC-PF algorithm has a better detection probability than the previous two algorithms, with a target presence probability below 40% for the first appearance and then increasing frame-by-frame. However, the overall stability of the algorithm is poor, and it performs poorly from frame 50 to frame 60 when the target disappears. The algorithm proposed in this paper also has a 1-frame delay when target 1, target 2, and target 3 first appear, but it still achieves more than 40% presence probability. In addition, this paper’s algorithm target presence probability is closer to the real presence probability of the target shown in Figure 4; the probability of the target’s presence in the case of multi-target reaches more than 80% and maintains better stability.

By comparing the target presence probability of the four algorithms in Figure 5 and Figure 6, respectively, it can be seen that the traditional PF algorithm and the FA-PF algorithm do not differ significantly from each other in coping with the case of the uniformly moving target and the case of the uniformly accelerated target. The STC-PF algorithm still outperforms the previous two algorithms in terms of detection probability, but the level of detection slips significantly when dealing with variable-speed targets, with a reduction of at least 10% in the average probability of the presence of multiple targets. The detection probability of the algorithm proposed in this paper is more stable, and the overall target presence probability remains above 80%.

### 5.3. Comparative Analysis of Root Mean Square Error

**Figure 7:**
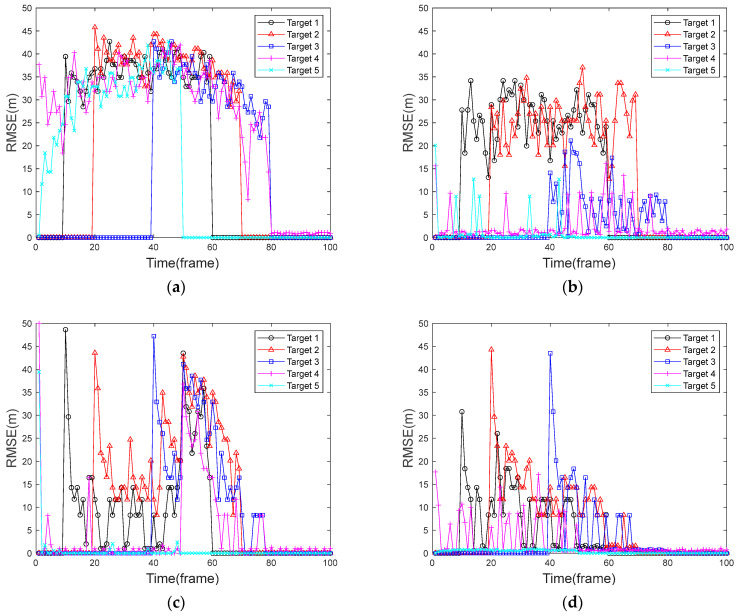
RMSE graph of a uniformly moving target. (**a**) Traditional PF algorithm; (**b**) FA-PF algorithm; (**c**) STC-PF algorithm; (**d**) Improved the PF algorithm.

**Figure 8:**
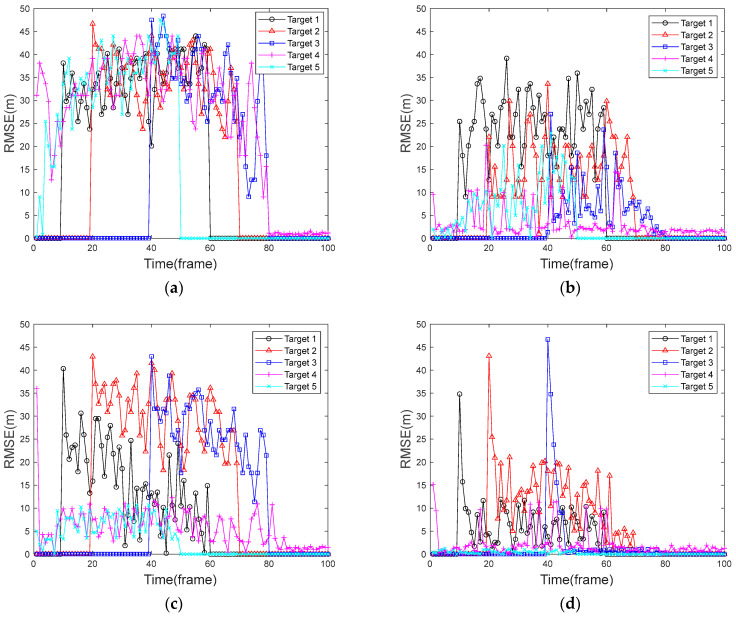
RMSE graph of a uniformly accelerated target. (**a**) Traditional PF algorithm; (**b**) FA-PF algorithm; (**c**) STC-PF algorithm; (**d**) Improved the PF algorithm.

By using the same experimental method as in Section 5.2, the RMSE is obtained for all targets in each frame, which in turn gives the average of 100 Monte Carlo simulation experiments. Figure 7 shows the RMSE in the case of a uniformly moving target, and Figure 8 shows the RMSE in the case of a uniformly accelerated target. Among them, Figure 7a and Figure 8a show the RMSE of the traditional PF algorithm, Figure 7b and Figure 8b show the RMSE of the FA-PF algorithm, Figure 7c and Figure 8c show the RMSE of the STC-PF algorithm, and Figure 7d and Figure 8d show the RMSE of the improved PF algorithm in this paper.

By comparing the target RMSE of the four algorithms in the context of a uniformly moving target in Figure 7, it can be seen that after the 80th frame, there is only one target phase; the RMSE values of the four algorithms are all within 3 m and are relatively stable. In the multi-target stage, the traditional PF algorithm has a considerable RMSE value for each target due to its very poor multi-target detection and tracking capability. The improvement of the FA-PF algorithm based on the traditional PF algorithm makes the algorithm have stronger multi-target detection and tracking ability, and the target RMSE values are much lower than that of the traditional PF algorithm. In particular, target 4 and target 5 that existed from the beginning generally have RMSE values within 10 m. Target 3 also obtained a lower error due to its proximity to the target 4.; The target RMSE values of the STC-PF algorithm increase abruptly at the first appearance of the target and then decrease frame-by-frame, but it is not guaranteed that the RMSE is at a low level for all the targets, and the average RMSE values of target 1, target 2, and target 3 are higher than 15 m. The algorithm proposed in this paper performs better in the multi-target case, where the RMSE values of the targets are gradually reduced to within 15 after the first detection, and the overall RMSE values are lower than those of the rest of the algorithms.

By comparing the RMSE of the four algorithms in Figure 7 and Figure 8, respectively, it can be seen that there is no significant difference between the traditional PF algorithm in coping with a uniformly accelerated target and uniformly moving target, and the RMSE values are all higher than 25 m. The ability of the FA-PF algorithm to deal with a uniformly accelerated target case decreases, and the target 5 error increases significantly. The STC-PF algorithm is substantially less stable in response to the uniformly accelerated target case and has a larger error than that in the uniformly moving target case. The algorithm in this paper is relatively stable, with overall RMSE values of less than 15 m for both the uniformly accelerated target case and the uniformly moving target case.

Table 3 shows the average value of RMSE for each target in the four algorithms for the two simulation scenarios. As can be seen from Table 3, in the target uniform motion mode, for target 4 and target 5, which existed at the beginning, the root-mean-square errors of the three algorithms other than the traditional PF algorithm have mean values within 5 m and do not show large differences. For the other three targets, the root-mean-square error average of this paper’s algorithm is much smaller than other algorithms and stays within 12 m.

When all targets are uniformly accelerated targets, the RMSE of the traditional PF algorithm for all five targets has an average value greater than 30 m. The mean RMSE values of the FA-PF algorithm changed more randomly compared to the target uniform motion mode. The STC-PF algorithm showed an overall significant increase in the mean RMSE values compared to the target uniform motion mode. The mean RMSE values of this paper’s algorithm are closest to the mean RMSE values of the target’s uniform motion mode, indicating that this paper’s algorithm is more adaptable in dealing with variable-speed targets. This performance is mainly due to the fact that this paper fully improves the particle diversity by combining two kinds of particles, and secondly, the optimization of the resampling process and state output process by the DBSCAN algorithm also makes the filtering results more accurate.

### 5.4. Comparative Analysis of the Effect of the Number of Targets on the Probability of Detection

**Figure 9:**
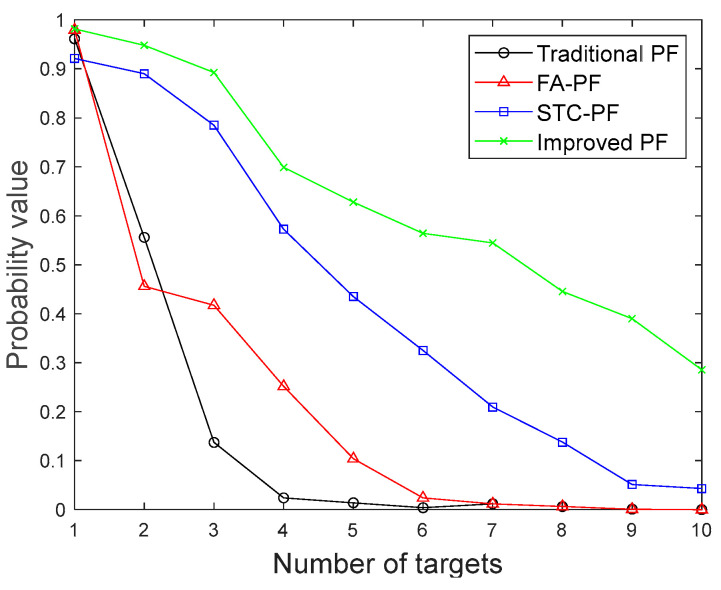
Comparison of the average probability of target presence among four algorithms.

In order to test the adaptability of each algorithm in dealing with different numbers of targets, simulation and comparison experiments are conducted for each algorithm under different numbers of targets. The number of targets set in the experiment is n, where n∈{1,2,3,4,5,6,7,8,9,10}. The setup targets are always present in the environment and are all uniformly moving targets. One hundred Mote-Carlo simulation experiments are performed for each experimental scenario to compare the average target presence probability of each algorithm. Figure 9 shows the average target presence probability of each algorithm for the different number of targets.

By comparing the average target presence probability of different algorithms in Figure 9, it can be seen that when the number of targets is 1, the average target presence probability of the four algorithms is relatively similar, all exceeding 90%; while the number of targets increased, the average probability of the presence of the four algorithms of the target gradually decreased. The algorithm in this paper still guarantees the average probability of target presence of more than 50% when the number of targets is 6. In the multi-target case, the algorithm presented in this paper consistently demonstrates the best performance among the four algorithms.

### 5.5. Computational Cost

In order to evaluate the real-time performance of the algorithms, the computational complexity of the four algorithms was tested separately. Three sets of complexity comparison experiments with target numbers of 1, 5, and 10 were selected. The setup targets always presented in the environment are all uniformly moving targets. The preprocessing steps in Section 5.1 were used to obtain 100 frames of data for the experiment, and the average time spent per frame was recorded. The average time consumption of each algorithm is calculated through 50 Mote-Carlo experiments. Table 4 shows the comparison of the time consumption of the four algorithms for different numbers of targets.

Then the computational complexity of the three phases of the traditional particle filter algorithm, state updating, weight calculation, and resampling, is O(N). So the computational complexity of the traditional particle filter algorithm is O(N). The resampling phase of the FA-PF algorithm requires comparing each particle with the rest of the particles, and the computational complexity of the resampling phase is increased to O(M × N^2). The STC-PF algorithm simplifies the multi-target case to multiple single-target cases, so the complexity of the algorithm increases dramatically as the number of targets increases. So the computational complexity of all three phases of the STC-PF algorithm is O(n × N). The clustering algorithm introduced in the improved algorithm of this paper makes the computational complexity of the resampling phase O(N^2). It can be seen from Table 4 that the traditional algorithm is the least time-consuming, whereas all three improved algorithms have different degrees of increase in terms of time consumption. The STC-PF algorithm takes the most time, and the improved algorithm in this paper is slightly less time-consuming than the FA-PF algorithm.

## 6. Conclusions

This paper proposes an improved particle filter algorithm for the problems of particle filter algorithm in the face of multi-target detection and tracking. This algorithm combines tracking particles and searching particles, optimizing the resampling step and state output process of the particle filter algorithm using the DBSCAN algorithm. This approach overcomes the difficulty of the existing detection and tracking algorithm based on the particle filter algorithm to deal with multi-target detection and tracking when the number of targets is unknown. It sufficiently improves the diversity of particles. After simulation experiments and comparisons, it is found that the improved algorithm effectively suppresses particle degradation, and at the same time, the detection probability and accuracy are better than similar algorithms.

## Figures and Tables

**Table 1:** Radar parameters.

Radar Parameters	Value
Radar radio frequency	1.57 GHz
Pulse repetition interval	1 ms
Number of pulses in a CPI	64
Range gate	10 m
Sampling frequency	10 MHz

**Table 2:** Target parameters.

Experiment	Serial Number	Target State	Occurrence Frame	Vanishing Frame
uniformly moving target	1	[2000 m, 200 m/s]	10	60
	2	[2800 m, 180 m/s]	20	70
	3	[3500 m, −200 m/s]	40	80
	4	[4000 m, −250 m/s]	1	100
	5	[5000 m, −150 m/s]	1	50
uniformly accelerated target	1	[2000 m, 200 m/s, −40 m/s^2^]	10	60
	2	[2800 m, 180 m/s, −25 m/s^2^]	20	70
	3	[3500 m, −200 m/s, 15 m/s^2^]	40	80
	4	[4000 m, −250 m/s, 30 m/s^2^]	1	100
	5	[5000 m, −150 m/s, −10 m/s^2^]	1	50

**Table 3:** Comparison of the average root mean square error of four algorithms.

Experiment	Serial Number	Average Root Mean Square Error			
		Traditional PF	PA-PF	STC-PF	Algorithm in This Paper
uniformly moving target	1	36.3322	32.9602	13.8739	8.3782
	2	38.4366	25.8424	23.5700	11.8662
	3	33.5475	7.4306	19.8356	7.0302
	4	25.3925	2.2511	4.3639	2.3374
	5	30.2542	1.6401	0.9788	0.6349
uniformly accelerated target	1	34.7787	24.9997	15.8337	6.7895
	2	35.0525	16.3058	30.5385	12.2005
	3	33.1559	7.9229	27.0117	3.9284
	4	25.5557	3.0871	5.7658	2.1686
	5	32.5426	7.8742	6.1259	0.5068

**Table 4:** Comparison of average time per frame for four algorithms.

Target Number	Traditional PF	PA-PF	STC-PF	Algorithm in This Paper
1	0.1166	0.1690	0.3250	0.1598
5	0.1179	0.1738	1.0962	0.1502
10	0.1193	0.1666	1.6251	0.1614

## Data Availability

Data are contained within the article.
